# Burial Duration and Frequency Influences Resilience of Differing Propagule Types in a Subtidal Seagrass, *Posidonia australis*

**DOI:** 10.1371/journal.pone.0161309

**Published:** 2016-08-15

**Authors:** Marnie L. Campbell

**Affiliations:** 1 School of Biological and Environmental Science, Murdoch University, Perth, Western Australia, Australia; 2 The Environmental Research Institute, University of Waikato, Hamilton, Waikato, New Zealand; Universita degli Studi di Genova, ITALY

## Abstract

Sedimentation that leads to periodic, and often prolonged, burial events is becoming more common on the world’s coastlines as human populations expand and create urbanised marine environments. Different seagrass species react differently to sediment burial but many species in the southern hemisphere are yet to be examined. How seagrasses react to burial has restoration implications. There is a need to critically assess seagrass transplant propagule responses to periodic (pulse) and prolonged (press) burial events before selecting the most appropriate species, transplant propagule, and transplant site. In my study, mesocosm experiments, coupled with field measurements were used to assess how sexual (seedlings) and vegetative (sprigs) propagules of *Posidonia australis* responded to pulse and press burial events. Seedlings were highly susceptible to burial (both pulse and press), with no survival at the end of the experimental period. In contrast, rhizome growth in vegetative propagules was stimulated by pulse burial, although press burial events resulted in mortality. The implication for *Posidonia australis* restoration efforts in areas where burial is periodic, was that vegetative propagules are optimal transplant units, in comparison to seedlings. Press burial however, renders a transplant site sub-optimal for both seedling and sprig transplants.

## Introduction

Success in seagrass restoration has proven to be highly variable (e.g., [1–3]) and influenced by a number of stressors (e.g., [4]). Successful transplantation and restoration (or rehabilitation) of seagrass rely upon knowledge of the target species’ autecological requirements to determine the appropriate transplant unit type [5,6] and appropriate recipient site [5], including understanding if site specific stressors that led to seagrass decline have been ameliorated or removed. Equally important is the knowledge of how the species responds to disturbance events such as sediment burial, intensity of herbivory, and changing nutrient levels (e.g., [7–14]). Consequently, restoration and rehabilitation success may be heavily influenced by disturbance at a proposed transplant site. Knowing how different seagrass propagules (sexual or vegetative) respond to stressors, particularly site specific disturbances, should drive decisions on the selection of both suitable transplant sites and transplant units.

Sediment deposition through natural means is associated with: normal riverine discharge; storm associated events such as wave resuspension, enhanced erosion and runoff; and inlet, sandbar and dune migration. These events typically result in short pulses of sediment load that is rapidly removed. In contrast, human alteration of land-based inputs [15, 16] has created enhanced sediment loads associated with watershed scale soil erosion, and alteration of sediment transport through diversion and reservoir retention schemes, resulting in global alterations of sediment delivery to coastal habitats [17]. Similarly, human activities such as coastal trawl fishing, dredging, dumping and construction alter sediment loads. Both natural and human generated activities result in short-term “pulse” events (which may be periodic) and prolonged “press” events (*sensu* [18]; but see [19]). Pulse and press events fall along a continuum; differentiation between these categories must be made relative to the observed organism or community or, as is used here, from the observed environment.

Studies have illustrated that burial triggers photomorphogenic responses in seagrasses that result in the relocation of the meristem to the surface (where possible; e.g., [20–21]). Typically, seagrass burial studies have examined depth of burial as the determinant of disturbance (e.g., [8, 11, 22–27]) but few have focussed on the duration or frequency of burial (except see [14,28]) or examined the resilience to burial between propagule types. These few studies have suggested that pulse versus press burial events may produce differing outcomes for seagrass resilience [11, 29–30]. For example, press burial events can cause seagrass declines (e.g., [31, 32]) and, contrary to ecological theory that pulse events would result in temporary effects, there is empirical evidence that some seagrasses are more likely to succumb to rapid pulse burial events [11, 14] and persist or thrive in press burial events [28, 29]. How seagrasses respond to pulse vs. press burial events remains largely unexplored, yet is critically needed to help select an appropriate seagrass transplant site and propagule type.

Exposure to burial events and the duration and frequency of repeating events are particularly important considerations for seagrass restoration ecologists. Transplant site selection is often driven by non-biological drivers such as economics, socio-political constraints and/or logistics, and may be physically sub-optimal based on the likelihood of burial disturbance. As a result, there is a need to critically assess species and transplant unit responses to pulse and press burial before selecting the preferred transplant site, the most appropriate species or transplant method.

In Western Australia, seagrass transplant efforts frequently fail (38% success rate; [2]), which has resulted in significant efforts to overcome problems associated with site specific stressors such as surge and wave exposure (e.g., [33–35]) through technical solutions (e.g. use of mats, anchors, barriers etc.). However, these technical solutions are resource intensive and rarely correct the long-term impacts. Here, I propose that instead of looking to technology to solve an issue, understanding the relationship between the impacts of pulse vs. press burial events and various transplant unit types (vegetative or sexual propagules) can inform future transplant efforts to improve success.

The current study investigated the resilience of *Posidonia australis* sexual (seedlings) and asexual (sprigs) propagules when exposed to varying burial durations. My hypothesis successfully tested if pulse and press burial events would limit horizontal rhizome growth. I have focussed on *P*. *australis* as it is a dominant seagrass across southern Australia, it is known to be susceptible to disturbance [36], and research groups are working to develop seagrass nurseries as restoration banks that include *P*. *australis* [37].

## Materials and Methods

Burial experiments were conducted in mesocosms to remove confounding factors that may have influenced the results if the study occurred in the field. Field control and mesocosm control measurements of rhizome growth were recorded to ensure that differences were due to treatment and were not mesocosm artefacts. All sampling occurred under a general permit (Western Australian Fisheries) that was issued to Murdoch University.

A total of 324 sprigs (vegetative propagules) and 540 fruits (sexual propagules) of *Posidonia australis* were collected from depths of 5 to 9 m on Success Bank (Perth, Western Australia; 32°05’94’ S; 115°43’94’ E). Vegetative propagules were sourced from an area that was later dredged. Upon collection, all vegetative propagules were tagged (using methods of [35]) and labelled (to maintain individual identification), washed in a disinfectant solution (1:80 chlorox:seawater) to reduce potential infection and then randomly allocated to outdoor, independent flowing seawater mesocosms (n = 18). Mesocosms had a capacity of 180L and an average flow rate of 240L h^-1^. The mesocosm laboratory was shaded to reduce light levels to within statistically similar levels (average 407.35 μmol m^-2^s^-1^) to those found at depths where the seagrasses were collected (5m on Success Bank; t_[36]_ = 1.26; p > 0.05). Each mesocosm received six vegetative propagules and 10 sexual propagules. Fruits were also washed in the disinfectant solution.

Vegetative (sprigs) propagules consisted of an apical meristem (to ensure potential rhizome growth) and at least five shoots. These were planted within the sediment to the depth observed in the field (e.g., meristems at the sediment surface). Fruits were allowed to dehisce and release seedlings. *Posidonia australis* seedlings are relatively large compared to seedlings of species such as *Halophila ovalis* and *Zostera tasmanica*. On average, the seedlings in this experiment were 3–4 cm in length when the experiment began. Once released, the seedlings were tagged (using a very small plastic cable tie on the radicle), labelled (using an alpha numeric code scratched into the cable tie) and planted as described above. After planting, a 30-day acclimation period occurred before the experimental treatments begun. Mesocosm irradiance averaged 407 μmol photons m^-2^s^-1^ and water temperature averaged 20°C throughout the experimental period. Photoperiod matched the natural, spring environment (12L: 12D). Mesocosms were randomly allocated to treatments and controls (seven pulse, seven press, and four controls) which are described further below.

Horizontal rhizome growth (extension) was measured, noting that *P*. *australis* does not produce vertical rhizomes, by tagging the last shoot on the rhizome prior to the apical meristem. An increase in net rhizome length beyond the tagged area indicated growth, while a reduction indicated necrosis. This method has been successfully employed on the congener *Posidonia oceanica* [38, 39], and on *P*. *australis* and *P*. *coriacea* in the field [1, 40].

Experiments were performed over three consecutive spring/summer periods between 1993 and 1995. No significant differences between the results in each of the replicates were detected, therefore all subsequent data is a mean of all experiments. Fresh propagule material was collected at the beginning of each experimental occasion.

### Control

Four mesocosms were established as controls, where propagules were planted during the acclimation period and remained undisturbed except for rhizome measurements at t = 0 (after the acclimation period), at day 43 (due to logistic constraints the day following the end of Treatment 2) and at day 63 (the end of Treatment 1). At day 43, 32 randomly selected propagules (12 vegetative and 20 sexual) from within all of the control mesocosms were removed and rhizome extension was measured. The remaining 32 propagules had rhizome growth measured at day 63.

A field control on Success Bank was established in a depth of 5 m for comparative purposes with 60 *Posidonia australis in situ* meadow rhizomes tagged and measured every three-months for 23 months (see [41]). Field light levels ranged from 321 μmol photons m^-2^ s^-1^ in summer to 144 μmol photons m^-2^s^-1^ during winter [1]. During the period of study, naturally recruiting vegetative fragments were observed at the field control site [40]. These naturally recruited vegetative fragments were tagged and measured *in situ*, in similar fashion to the field control.

### Treatment 1: Pulse Burial

Seven mesocosms were randomly selected for Treatment 1, which simulated periods of burial observed in the field (average burial duration of 21 days; [1, 41]). This treatment represented a pulse event where the burial effect was cyclical. Propagules within these mesocosms were covered with acid-washed sand so that no leaves were left exposed (~ 4 cm depth). This depth was selected as it reflected the average deposition levels observed in the natural environment of this region (discussed in [1, 41]). Sediment deposition in this region is driven by the physical environment (storm and wind driven waves) [1, 41]. After 21 days burial, sand was gently fanned away to uncover the propagules allowing leaf exposure to light. Rhizome extension was measured at this time and propagules were left uncovered for a further 21 days, simulating a ‘recovery’ period. Rhizome extension was then measured and the propagules re-covered with sand for a further 21 days resulting in a total burial duration of 42 days (roughly equivalent to Treatment 2 duration). The treatment was then terminated and final rhizome lengths were measured.

### Treatment 2: Press Burial

Seven mesocosms were established for Treatment 2, which simulated a prolonged, or press, burial event at durations similar to those that occurred in the field [1, 41]. Burial of propagules so that no leaves were left exposed (~ 4 cm depth) occurred in the same manner as in Treatment 1, with the exception that propagules were left covered for the entire period of 43 days. The treatment was then terminated without any ‘recovery’ period and final rhizome lengths were measured.

### Statistical Analyses

A one-way ANOVA (*p* = 0.05) was used to detect differences in propagule response (rhizome growth) to experimental burial duration (pulse and press). If a statistically significant difference was detected, post-hoc all-pairwise analyses were used to further elucidate the statistical differences. Treatment controls were compared against field controls in order to demonstrate similar conditions. Subsequently, all mesocosm treatments (pulse and press experiments) were compared against the mesocosm control. Naturally recruiting vegetative fragments were compared against field controls.

## Results

### Sexual propagules (seedlings)

#### Control and field outcomes

Extensive flowering was observed in the field. However, seedling recruits were not detected over the three years that the experiments ran, although widespread searches were undertaken. Consequently, no seedling growth data in the field was collected. Failure of *P*. *australis* seedling recruitment and establishment in the field has been demonstrated previously despite the seed’s high viability and production [40].

In general, 89% of mesocosm seedlings produced a small rhizome, however these did not grow appreciably (<1 mm over the entire experimental period) or only produced root hairs. Fewer than three leaves were present on 98% of the seedlings at the end of the acclimation period. All seedlings in the control mesocosms survived but no rhizome extension occurred during the experimental periods. No seedlings survived either beyond the first burial period in Treatment 1 or at the end of Treatment 2, suggesting a high vulnerability to both pulse and press burial disturbance.

### Vegetative propagules (sprigs)

#### Control and field outcomes

**Fig 1:**
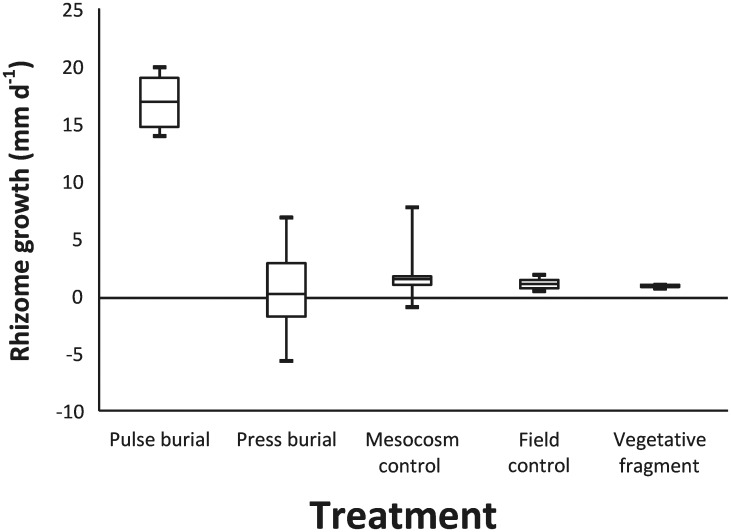
Box plot of *Posidonia australis* net rhizome extension rates (mm d^-1^) by treatment. The central solid line represents the median (50% Quartile); the top and bottom of the box represent the 25% and 75% Quartiles, respectively; and the vertical bar represents the observed range of maximum and minimum values.

In the field, control vegetative propagules grew at a rate of 1.0 ± 0.09 mm d^-1^, while vegetative propagules within the mesocosm controls grew at an average of 1.7 ± 0.41 mm d^-1^ (Fig 1). Comparison of rhizome growth rates between field and mesocosm controls were not statistically different (t_[20]_ = 1.66, p = 0.113). Naturally recruiting vegetative fragments in the field grew at a rate of 0.78 ± 0.02 mm d^-1^ [40] and exhibited significantly lower rhizome extension rates than the field control (t_[20]_ = 2.43, p = 0.025; Fig 1).

#### Treatment 1: Pulse burial

Vegetative propagules grew an average of 16.7 ± 0.84 mm d^-1^ throughout the 63 day experimental period (Fig 1). Rhizome growth significantly differed between the pulse treatment and the mesocosm control (t_[9]_ = 16.04, p = 6.27*10^−8^), with rhizome growth being greater in the pulse burial treatment (Fig 1).

#### Treatment 2: Press burial

Vegetative propagules exposed to press burial exhibited low rhizome extension rates (0.13 ± 1.0 mm d^-1^, Fig 1). In general however, exposing rhizomes to press burial resulted in necrosis of all vegetative rhizomes. Rhizome growth was significantly lower under press burial than in the mesocosm control (t_[15]_ = 1.45, p = 0.169) and also significantly lower than those exposed to cyclical pulse burial (t_[17]_ = 12.62, p = 4.62*10^−10^; Fig 1).

## Discussion

Global seagrass loss has created significant alterations of coastal ecosystems leading to numerous calls for management through restoration (e.g., [42, 43]). Many available sites for restoration efforts are in exposed and typically high sediment load, and therefore accreting, environments (e.g., [35, 44–45]); concomitantly, seagrasses create an accreting ecosystem (e.g., [46]). Therefore, the rate and duration of burial is potentially a significant determinant of restoration success, particularly if the resilience to burial varies between species or transplant unit types (e.g., [14, 28, 47]). This study found that resilience to burial duration for *Posidonia australis* varied between transplant unit types. Seedlings (< 93 days old) are highly susceptible to both pulse and press burial events. Growth of *P*. *australis* rhizomes in sprigs were stimulated by pulse burial events, but succumbed to press burial events of up to 43 days (burial periods emulated field conditions; [1, 41]). These findings clearly demonstrate that *P*. *australis* sprigs should be the preferred transplant unit type in sites where cyclical pulse burial events occur, but noting that longer-term press burial events are detrimental to both seedlings and sprigs. The specific trade-off between cyclical pulse, versus press events remains a critical area of research.

The increasing anthropogenic influences in expanding urbanised coastal systems [48], such as coastal dredging and land run off, often result in sedimentation pulses (e.g., [32]) leading to an increased frequency and duration of burial events [49]. These sediment pulses may also bring catchment pollutants (e.g., [50]). Furthermore, frequency of sedimentation is likely to increase with climatic changes in some regions (e.g., [51, 52]), again resulting in increased burial risk for seagrasses (e.g., [53]) and potential cumulative effects from catchment pollutants (e.g., [50]).

The finding of increased rhizome growth under pulse burial conditions is not unusual. Experimental studies of species with vertical rhizomes have demonstrated the relocation of the apical meristem towards light and increased vertical shoot growth when moderately buried (e.g., [8, 24–26, 54]). This study however, has demonstrated that *P*. *australis* plants which do not have vertical shoots, still exhibited a significant stress response by investing in rhizome growth as a mechanism to relocate the meristem to the surface. Photomorphogenic responses in seagrasses have been reported or hypothesised in relation to self-thinning [55], rhizome internode elongation and branching [56, 57], burial [20] and carbohydrate stores [14]. Similarly, Marbà and colleague [58] identified variability in rhizome growth due to variations in space availability between patch edges and centres.

Results from this study indicate that a slower-growing species, such as *P*. *australis*, can respond to pulse burial in a fashion similar to smaller, faster-growing species such as *Cymodocea nodosa*. Researchers have hypothesised that some dominant, slower-growing species, such as *Phyllospadix scouleri* and *Posidonia oceanica*, can withstand burial because of their rhizomatous root system [23, 59] that provides a carbohydrate buffer to stress. Yet, some dominant, slow-growing species, such as *C*. *serrulata* [8] and *Zostera* species [60], do not respond rapidly to sedimentation and hence do not survive press burial events (e.g., [14, 29]). “Slower-growing” species are not opportunistic in their reaction to burial or disturbance; they can be stress-tolerant at first [59], but if burial is prolonged they may succumb [8, 14, 29, 59]. This appears to be the case for *P*. *australis* in this study. Under pulse burial stress, rhizome growth increased to more than 16 times that observed in the field. However, propagules eventually succumb to press burial stress (Fig 1).

*Posidonia australis* seedlings were extremely susceptible to both pulse and press burial, with complete mortality during the experimental period. This rapid response to burial was unexpected, as *P*. *australis* seedlings reportedly have a seed cotelydon capable of providing enough stored carbohydrates to survive for nine months [61, 62]. Mortality would be expected if a seedling had not grown leaves and replenished carbohydrate stores in that timeframe. The experimental period used here was one quarter of this timeframe and it is unlikely that carbohydrate stores had been depleted (although this parameter was not measured here), yet mortality in both pulse and press burial treatments was observed.

Seed coat light interactions may explain the outcome of this study. Light has an integral role in controlling the growth response in some buried seagrass seedlings [20]; a lack of light will stimulate growth and the presence of light deters it. This pattern is evidenced by a reduction in rhizome growth when plants are in an eroding environment [63–64]. Yet, in my study the lack of light failed to stimulate growth of *P*. *australis* seedlings. Photosynthetic activity in the seed coat of *P*. *oceanica*, *P*. *australis*, *and P*. *sinuosa* enhances seedling growth [65]. Thus, burial events may have disrupted the seed photosystem potential and have led to the high mortality of seedlings in both pulse and press treatments in this study.

### Implications to restoration

This study’s findings have a number of explicit implications for *P*. *australis* restoration efforts, and potentially other ‘climax’ seagrass species. The long-standing and currently accepted paradigm for seagrass transplant efforts considers that previously occupied sites reflect realised niches (e.g., [66–70]) and will therefore provide the most suitable conditions for transplantation. This model is counter to conservation biology theory that suggests that once a site deviates from its original trajectory, returning to this trajectory is unlikely, if not implausible (also referred to as the ‘Humpty Dumpty’ rule; [71]). This observation has recently entered the seagrass literature (e.g., [72]). Furthermore, if previously occupied sites are in high energy conditions (i.e., exposed offshore environments) where seagrass transplant efforts experience high water movement and frequent burial, then success may be limited (e.g., [34, 44, 45, 73]) regardless of whether the changed system could be rehabilitated.

The creation of land-based nurseries to grow transplant propagules (e.g., [37]) should factor in the rate at which seedlings succumbed to burial stress. Thus, investment in a nursery system should determine at what age or size (e.g., [36]) seedlings become sufficiently robust to survive being transplanted into conditions that are subject to sedimentation. If seedlings can be cultivated to a large size equivalent to a ‘sprig’, then these transplant units are more likely to survive pulse burial events but not press burial events (>21 days). The lack of evidence of seedling establishment in the field, coupled with observations of vegetative fragment recruitment of *P*. *australis* [35] supports the inference that seedlings need to be of a robust size to survive transplantation.

In conclusion, my results demonstrate that *P*. *australis* rhizome extension was stimulated when burial was pulsed, but decreased under press burial conditions. This indicates that this species is stress tolerant to a point. Conversely, *P*. *australis* seedlings were highly susceptible to both pulse and press burial. These results suggest that *P*. *australis* vegetative propagules are more resistant to burial than *P*. *australis* seedlings and hence, vegetative propagules are an optimal transplant unit for areas where burial events occur. Understanding how an individual species and its propagules are influenced by burial in press and pulse situations is a critical consideration when attempting to transplant and restore seagrasses.
